# Mechanisms controlling hormone secretion in human gut and its relevance to metabolism

**DOI:** 10.1530/JOE-19-0399

**Published:** 2019-11-18

**Authors:** Alyce M Martin, Emily W Sun, Damien J Keating

**Affiliations:** 1 College of Medicine and Public Health, Flinders University, Adelaide, South Australia, Australia; 2 Nutrition and Metabolism, South Australian Health and Medical Research Institute, Adelaide, South Australia, Australia

**Keywords:** GLP-1, PYY, serotonin, CCK, GIP, obesity, diabetes, microbiome, liver, pancreas, adipose tissue

## Abstract

The homoeostatic regulation of metabolism is highly complex and involves multiple inputs from both the nervous and endocrine systems. The gut is the largest endocrine organ in our body and synthesises and secretes over 20 different hormones from enteroendocrine cells that are dispersed throughout the gut epithelium. These hormones include GLP-1, PYY, GIP, serotonin, and CCK, each of which play pivotal roles in maintaining energy balance and glucose homeostasis. Some are now the basis of several clinically used glucose-lowering and weight loss therapies. The environment in which these enteroendocrine cells exist is also complex, as they are exposed to numerous physiological inputs including ingested nutrients, circulating factors and metabolites produced from neighbouring gut microbiome. In this review, we examine the diverse means by which gut-derived hormones carry out their metabolic functions through their interactions with different metabolically important organs including the liver, pancreas, adipose tissue and brain. Furthermore, we discuss how nutrients and microbial metabolites affect gut hormone secretion and the mechanisms underlying these interactions.

## Introduction

Enteroendocrine (EE) cells are specialised hormone-secreting cells that are dispersed throughout the mucosal epithelial layer of the gastrointestinal (GI) tract. Collectively, these cells constitute 1% of the mucosal cell population and are, by mass, the largest endocrine tissue in the body (Ahlman & Nilsson 2001). EE cells consist of an array of different cell types, synthesising and secreting a combination of more than 20 hormones in response to a variety of luminal and basolateral stimuli. The characterisation of distinct EE cell types has been traditionally based on their dominant and supposedly unique hormone expression profile, such as enterochromaffin (EC) cells secreting serotonin (5-HT), L cells secreting glucagon-like peptide 1 (GLP-1), peptide YY (PYY) and oxyntomodulin (OXM), and glucose-dependent insulinotropic peptide (GIP) secreting K cells. It is now clear that such a classification system is not accurate given the accumulation of evidence that cross-over in hormone co-expression exists in a regionally distinct manner, giving rise to an array of EE cell subtypes (Fothergill* et al*. 2017).

Gut-derived hormones influence a range of physiological processes, including metabolic pathways. They perform these regulatory roles in glucose homeostasis, centrally-mediated appetite control and adiposity. This review focuses on the molecular mechanisms driving gut hormone secretion and describes how this is significant in the context of human metabolism and the pathogenesis of human metabolic disorders.

## Gut hormone regulation of metabolism

**Figure 1:**
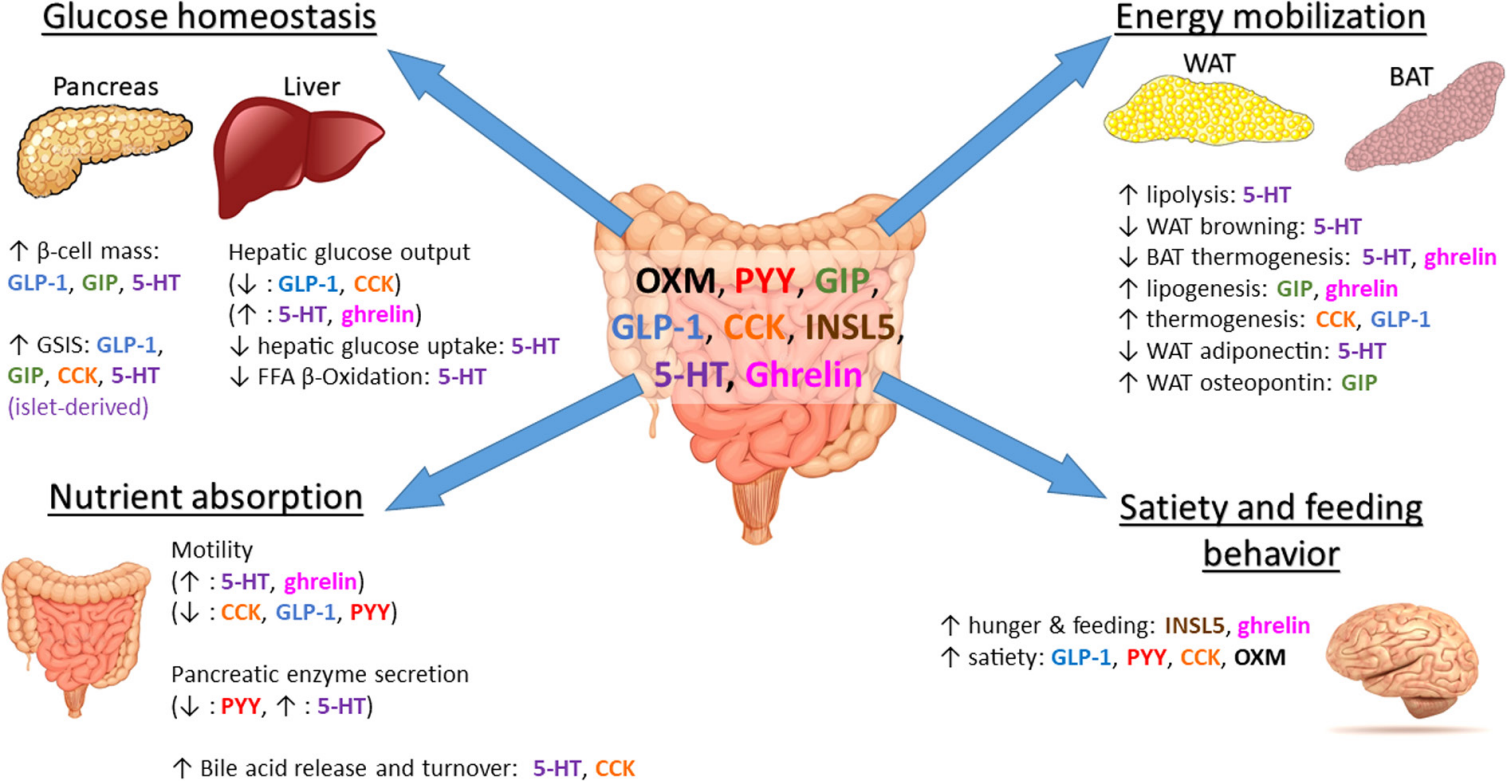
The role of gut hormones in metabolically important organs. Gut hormones are implicated in the regulation in glucose homeostasis through their differential actions on the liver and endocrine pancreas. They also play important roles in maintaining energy balance by modulating nutrient absorption, mobilization of fat stores from adipose tissue and appetite regulation. 5-HT, serotonin; CCK, cholecystokinin; GIP, glucose-dependent insulinotropic peptide; GLP-1, glucagon-like peptide 1; INSL5, insulin-like peptide 5; PYY, peptide YY; OXM, oxyntomodulin; BAT, brown adipose tissue; WAT, white adipose tissue.

The regulation of whole-body metabolism involves the integrated activity of multiple metabolically active tissues, including the GI tract, pancreas, adipose tissue, liver and the central nervous system (CNS). The release of one or a combination of gut hormones either postprandially (GLP-1, GIP, PYY, 5-HT, CCK, OXM) or during periods of fasting (ghrelin, 5-HT) significantly influences both glucose homeostasis and overall energy status. Each of these hormones can exert such effects independently or can act in a synergistic manner to influence these processes (Fig. 1).

### Glucose homeostasis

The coordinated control of endogenous glucose output and the clearance of exogenous glucose is required to maintain blood glucose homeostasis. If not adequately controlled, this can lead to metabolic diseases such as type 2 diabetes (T2D) and markedly increased cardiovascular risk. Hepatic glucose production is the primary determinant of glucose homeostasis and is predominantly dictated by pancreatic insulin and glucagon. In addition, postprandial glucose disposal by other insulin-sensitive tissues such as skeletal muscles and adipose tissue, and exogenous glucose uptake by the intestine also significantly determines peripheral glucose levels. In particular, the distal small intestine has been shown to contribute to gastrointestinal-mediated glucose disposal in both healthy and type 2 diabetic humans and coincides with incretin hormone release (Zhang* et al*. 2019). Gut hormones have well-established glucoregulatory roles, via activation of receptors expressed by target tissues.

#### Endocrine pancreas

Dysregulated secretion of insulin and glucagon, the two primary islet hormones, is a major contributing factor of the development of diabetes mellitus. Although a number of gut-derived hormones augment glucose-stimulated insulin secretion (GSIS) from pancreatic β cells and expansion of β-cell mass, some of these including GLP-1, GIP, CCK, PYY and 5-HT, have also been identified in the pancreas (Fujita* et al*. 2010). As such, the relative contributions of islet- and gut-derived hormones to pancreatic function have been increasingly scrutinised. It was originally thought that enteroendocrine L cells and K cells release GLP-1 and GIP, respectively, in response to intraluminal glucose and these two peptides then stimulate pancreatic β cells in an endocrine manner. While β cell GLP-1R have been consistently demonstrated to be essential for maintaining glucose homeostasis (Smith* et al*. 2014, Grasset* et al*. 2017), the physiological ligand for β cell GLP-1R remains a subject of controversy. Small amount of GLP-1 has been demonstrated to be produced by pancreatic islets (Marchetti* et al*. 2012) and Chambers *et al*. reported glucose tolerance in mice was significantly impaired by islet-, but not intestinal-specific ablation of *Gcg* (encodes of proglucagon derived peptides that include glucagon, GLP-1, GLP-2 and oxyntomodulin) (Chambers* et al*. 2017), supporting the notion that it is islet-derived, in a paracrine manner, rather than gut-derived GLP-1, in an endocrine fashion, that is crucial for gluco-regulation. However, this view has been challenged by several recent findings. Firstly, other groups have reported that only an extremely small amount of GLP-1 is produced by islet under normal physiological conditions (Song* et al*. 2019). Moreover, β cell GLP-1R is activated by glucagon at levels observed within islet microenvironments (Svendsen* et al*. 2018). As such, one can no longer conclude that islet-derived GLP-1 is essential for glucose homeostasis based on experiments in which a GLP-1R antagonist was used or involved site-specific *Gcg* knockdown, since pancreatic glucagon and GLP-1 actions are both blocked in these experiments. The necessity of gut-derived GLP-1 in glucoregulation is further complicated by a recent report showing that when *Gcg* is ablated specifically in the mouse ileum and colon, both oral and intraperitoneal glucose tolerance are significantly impaired, despite a compensatory upregulation of GIP (Song *et al*. 2019), landing support to the notion that gut-derived GLP-1 does play an essential role in gluco-regluation. Pancreatic β-cells also synthesise and secrete 5-HT, and this is important for the pregnancy-induced expansion of β-cell mass that occurs during pregnancy and which is essential to avoid gestational diabetes (Kim* et al*. 2010).

#### Liver

The liver is central in maintaining glucose homeostasis with hepatic glucose output through glycogenolysis and gluconeogenesis being the biggest contributor to plasma glucose levels in the post-absorptive state (Sherwin 1980). During the postprandial period, hepatocytes increase glucose uptake and upregulate glycogen synthesis in response to elevated insulin and reduce glucose output in response to decreased glucagon levels. Dysregulated suppression of hepatic glucose output during the postprandial period is a major driver of postprandial hyperglycaemia in T2D patients (DeFronzo* et al*. 1989). Several gut-derived hormones contribute to hepatic glucose output through their capacity to augment hepatic gluconeogenesis and glycogenolysis. In addition, hepatic insulin clearance has been recently demonstrated to contribute significantly to insulin action, through controlling insulin availability to peripheral tissues (Bojsen-Moller* et al*. 2018).

Postprandial release of GLP-1 attenuates hepatic glucose production, independent of its effects on pancreatic islets (Jun* et al*. 2015), potentially through activation of GLP-1R on vagal afferent nerves innervating the hepatic portal vein (Vahl* et al*. 2007). CCK suppresses hepatic glucose output in rodents by acting on CCK-A receptors on intestinal vagal afferents projecting to the nuclear solitary tract (NTS), a signalling pathway that is perturbed by diet-induced obesity (Cheung* et al*. 2009). However, whether such mechanism exist in humans remains a subject of contest. Various *in vivo* studies indicated that although CCK infusion lowers postprandial plasma glucose levels in humans, this is likely to be mediated by its inhibitory effect on gastric emptying (Liddle* et al*. 1988, Fried* et al*. 1991) or potential insulinotropic actions (Ahren* et al*. 2000), rather than direct effects on the liver. Opposingly, the release of gut-derived 5-HT during fasting increases hepatic gluconeogenesis and glycogenolysis while inhibiting peripheral glucose uptake (Sumara* et al*. 2012). Perhaps the most well-known of the gut-derived hormones released during fasting is ghrelin, with intraduodenal infusion increasing hepatic glucose production via GSH-R1a receptors on vagal afferents signalling to the NTS (Lin* et al*. 2019).

### Energy balance

Energy homeostasis is maintained through a delicate balance between calorie intake and expenditure, which ultimately determines bodyweight. Energy intake is determined by feeding drive and efficiency of nutrient absorption while energy expenditure is primarily governed by energetic cost to maintain basic cellular metabolic processes, thermal regulation and voluntary movements such as exercise. In the next section, we review how gut hormones influence energy balance through their effects of these governing factors.

#### Satiety and feeding behaviour

Gut-derived hormones play an integral role in appetite regulation, which in turn governs food intake, one of the major pillars of maintaining energy balance. The anorexigenic gut hormones GLP-1, PYY, CKK and OXM are released postprandially to induce satiety and reduce food intake whilst levels of the orexigenic hormones ghrelin and INSL5 are elevated during fasting to induce hunger and drive feeding behaviour (Sun* et al*. 2018). Gut hormones released by EECs stimulate vagal afferent nerve fibres by activating receptors located on nearby nerve endings, which project to appetite control nuclei of the brainstem. The anorectic effects of GLP-1 and CCK are significantly attenuated in vagotomised patients (Joyner* et al*. 1993, Plamboeck* et al*. 2013) whilst ghrelin receptors on gastric afferent nerve terminals mediate ghrelin-induced feeding (Date* et al*. 2002). Diet-induced obesity disrupts this neuroendocrine signalling pathway between the gut and the brain, dampening the activity of anorexigenic hormones and causes hyperphagia (de Lartigue 2016).

Gut hormones can also carry out appetite regulatory effects in an endocrine fashion where, via the fenestrated capillaries, circulating hormones reach the arcuate nucleus (ARC) in the hypothalamus (Cone 2005) as well as the NTS and area postrema (AP) in the brainstem (Murphy & Bloom 2004). Together, these form the key appetite centres in mammals. Within the ARC, the orexigenic AgRP/NPY neurons are activated during fasting and drive acute food seeking and consumption (Aponte* et al*. 2011). Ghrelin potently activates AgRP neurons while 5-HT, CCK and PYY, hormones that are released postprandially, all suppress AgRP neuron activity (Beutler* et al*. 2017). Acute food intake rapidly inhibits AgRP neuron firings (Beutler *et al*. 2017), resulting in the disinhibition of neighbouring anorexigenic proopiomelanocortin (POMC) neurons. Contrasting the rapid but short-lived effects of AgRP neurons, activation of POMC neurons in the ARC reduces food intake in a delayed but more sustained manner (Aponte *et al*. 2011). The GLP-1R agonist, liraglutide, acts on GLP-1R on ARC POMC neurons to reduce food intake and protect mice from diet-induced obesity (Burmeister* et al*. 2017). Within the brainstem, neurons of the NTS and adjacent AP are activated by gut-derived satiety signals primarily via the sensory vagus nerve and to a lesser extent by circulating gut-derived hormones. Similar to neurons within the ARC, the NTS and AP produce both NPY and POMC, and have reciprocal connections with the ARC that allows for extensive communication between the brainstem and hypothalamus to regulate feeding behaviour (Wynne *et al*. 2005). This central appetite-regulating pathway is made even more complex by the ability of sensory inputs arising from food detection to reverse orexigenic signalling *in vivo* in mice prior to food consumption. Intriguingly, the magnitude of response to food detection is also dictated by the hedonic properties of the food itself, such as palatability and energy density (Chen *et al*. 2015). Whether this is also driven by gut-derived hormones in response to potential olfactory cues is unknown.

#### Nutrient absorption

GI motility heavily influences the digestion and absorption of nutrients across the gut lumen, whereby contributing to glucose homeostasis and overall energy intake. A relationship exists between gastrointestinal motility and glycaemic control, as the rate of gastric emptying heavily influence oral glucose absorption and hence, postprandial glucose excursion (Rayner* et al*. 2001). CCK, ghrelin, PYY, GLP-1 and 5-HT are potent stimulators of the ENS to modulate GI motility. EC cell-derived 5-HT increases the frequency and force of colonic contractions (Keating & Spencer 2010). Ghrelin stimulates gastric motility and as such accelerates gastric emptying, alleviating the sense of fullness caused by gastric distention (Muller* et al*. 2015). Conversely, most of the anorexigenic gut hormones inhibit GI motility. CCK inhibits gastric emptying by mediating vasoactive intestinal peptide-induced relaxation of the gastric fundus as part of a vago-vagal reflex pathway (Grider 1994). GLP-1 delays gastric emptying and potently suppresses small intestinal motility (Hellstrom* et al*. 2008), an effect that is ascribed to some of the glucose-lowering effect of the GLP-1R agonist, liraglutide (Nakatani* et al*. 2017). Further to this, another GLP-1R agonist, exenatide, has been demonstrated to clinically suppress small intestinal motility and glucose absorption rate in both healthy individuals and those with type 2 diabetes (Thazhath* et al*. 2016). Similarly, the other L cell hormone PYY inhibits proximal intestinal motility, as part of an ‘ileal break’ mechanism (Maljaars* et al*. 2008).

Optimal nutrient absorption is heavily reliant on efficient digestion of ingested foodstuff, a process that is regulated by gut hormones. CCK is the major gut hormone that triggers gallbladder contraction and exocrine pancreatic secretion. The former releases bile acids, amphiphilic molecules that aid the solubilization of luminal lipids, whilst the latter consists of a mixture of digestive enzymes such as lipase, amylase and proteases, critical for the breakdown of macronutrients. Secretin released from the proximal small intestine following exposure to prandial gastric acid also stimulates secretion of pancreatic digestive enzymes and biliary bicarbonate secretion, while also reducing gastric emptying and gastric acid secretion (Afroze* et al*. 2013). EC cell-derived 5-HT is also implicated in the secretion of pancreatic enzymes and bicarbonate from the exocrine pancreas (Li* et al*. 2000, 2001), the latter being crucial in neutralizing gastric acid that would otherwise denature the enzymes (Keller & Layer 2005). An inhibitory role of PYY on exocrine pancreatic secretion had been suggested (Tatemoto 1982, Jin* et al*. 1993), although this could be mediated by pancreatic islet-derived PYY in a paracrine manner (Shi* et al*. 2015). While GLP-1 does not appear to directly affect gallbladder motility (Smits* et al*. 2016), there is evidence in support of a modulatory role to antagonize CCK-induced gallbladder contraction (Keller* et al*. 2012). On the other hand, a recent study showed that GLP-2, another L cell hormone, induces gallbladder relaxation and promotes gallbladder refilling (Yusta* et al*. 2017).

#### Energy mobilization

Adipose tissue exists as two subtypes: white adipose tissue (WAT) and brown adipose tissue (BAT), each serving distinct metabolic functions. The body’s surplus energy is primarily stored in WAT as triglycerides and liberated from the adipocyte as free fatty acids and glycerol when required. Excess adiposity secondary to increased fat storage within WAT is a key driver of obesity. On the other hand, BAT is implicated in thermal adaptation through its thermogenic capacity, dissipating energy harvested from the proton gradient across the inner mitochondrial membrane as heat, instead of coupling to ATP production (Bouillaud* et al*. 1983). Under certain conditions such as cold exposure, WAT has the capacity to undergo ‘browning’, whereby the expression of key genes controlling thermogenesis, particularly *UCP1*, are upregulated and cells transform towards those of a thermogenic phenotype resembling that of BAT (Bartelt & Heeren 2014). Increasing WAT browning and expanding BAT volume have both been intensely investigated for their anti-obesity potential as both approaches favour the removal of the body’s excess energy stores by increasing heat production (Bartelt & Heeren 2014). Moreover, a recent study has suggested that elevated hypothalamic temperature secondary to BAT-mediated thermogenesis induces satiety by activating POMC neurons in the ARC (Li* et al*. 2018).

Several gut hormones exert their metabolic effects by targeting adipose tissue, differentially influencing the uptake, utilization and storage of lipids. Many of the obesogenic effects of 5-HT is mediated through its action on adipocytes. Whilst 5-HT potently stimulates lipolysis in WAT to release free fatty acids and glycerol, it impairs β oxidation in the liver and WAT (Rozenblit-Susan* et al*. 2016), preventing these tissue from utilizing the newly available free fatty acids. Moreover, 5-HT reduces energy expenditure by preventing WAT browning (Crane* et al*. 2015). Together with ghrelin, the two hormones downregulate the thermogenic capacity of BAT (Muller *et al*. 2015), thereby increasing energy conservation. In addition, together with GIP, ghrelin increases the storage of lipids by upregulating lipogenesis (Getty-Kaushik* et al*. 2006, Theander-Carrillo* et al*. 2006). On the other hand, gut hormones that increase thermogenic capacity of adipocytes can prevent the development of obesity. In rodents, CCK and GLP-1 are both implicated in diet-induced thermogenesis in BAT, as they activate vagal afferents which in turns results in increased sympathetic output to BAT (Blouet & Schwartz 2012, Beiroa* et al*. 2014). The role of GLP-1 in thermogenic capacity in humans is less clear, however, as several clinical trials with acute exposure to the GLP-1R agonists, liraglutide and exenatide, yield no differences in resting energy expenditure, while prolonged exposure to these agonists increased energy expenditure (Maciel* et al*. 2018). A recent study showed that the intestinal hormone secretin potently induces prandial BAT thermogenesis, independent of sympathetic activity (Li *et al*. 2018).

In addition to its critical role in energy storage and thermal regulation, adipose tissue is a prominent regulator of peripheral metabolism through the secretion of a range of adipocyte-derived hormones, termed adipokines. Gut hormones have the capacity to alter the release of these adipokines, which poses another secondary mechanism by which they regulate peripheral metabolism via adipose tissue. Specifically, 5-HT attenuates the release of adiponectin from WAT (Uchida-Kitajima* et al*. 2008), an insulin-sensitizing, anti-lipogenic and anti-atherogenic adipokine (Stern* et al*. 2016). GIP upregulates the expression and stimulates the secretion of osteopontin, a pro-inflammatory adipokine derived from WAT that is implicated in the development of obesity-induced insulin resistance (Kiefer* et al*. 2010).

## Mechanisms controlling gut hormone secretion

**Figure 2:**
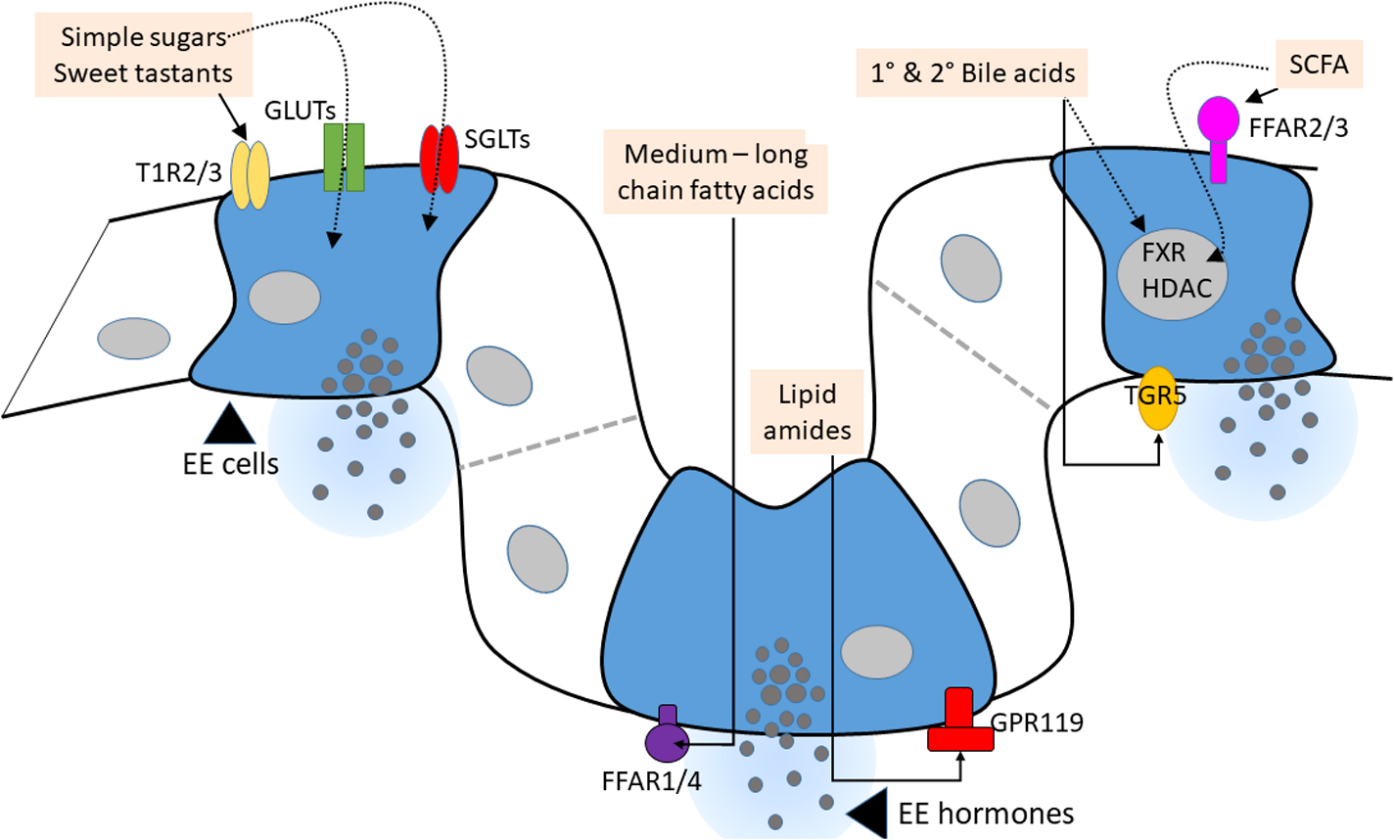
Activation of enteroendocrine (EE) cells. Carbohydrates in the gut lumen such as simple sugars are sensed by sodium-glucose transporters (SGLTs), glucose transporters (GLUTs) and sweet taste receptors (T1R2/3). Lipids are sensed basolaterally, with medium- and long-chain fatty acids activating free fatty acid receptors (FFARs) 1 and 4 and lipid amides activating G-protein receptor 119 (GPR119). EE cell secretion is also differentially regulated by individual bile acids, which signal through the GPCR, Takeda G-protein receptor 5 (TGR5) on the basolateral membrane and through the nuclear receptor, Farnesoid X receptor (FXR). Short-chain fatty acids (SCFAs) derived from bacterial fermentation of indigestible polysaccharides, also influence EE cell secretion through activating FFARs 2 and 3 and inhibiting histone deacetylases (HDACs).

Enteroendocrine cells are dispersed throughout the gut epithelium as individual cells. This makes it inherently difficult to isolate and study pure EE cells in culture and, as such, our knowledge on the mechanisms controlling gut hormone secretion has been predominantly derived from cell lines, or *ex vivo* and *in vivo* animal models. Recent advances in EE cell purification (Reimann* et al*. 2008, Martin* et al*. 2017*a*, Lund* et al*. 2018), particularly the availability of transgenic mouse lines in which specific EE cell populations can be fluorescently labelled and sorted (Reimann *et al*. 2008), have provided valuable insights into our understanding of the molecular mechanisms controlling gut hormone secretion. Functional transcriptomic and proteomic analysis of purified EE cells has revealed that these cells are sensors for an array of luminal nutrients and microbial metabolites (Fig. 2).

### Nutrient sensing

#### Carbohydrate

Most EE cell types have the capacity to sense luminal carbohydrates. During a standard oral glucose tolerance test, ghrelin secretion is significantly suppressed (Muller *et al*. 2015), whilst 5-HT, CCK, GIP and GLP-1 are increased (Gribble & Reimann 2016). Glucose-induced GLP-1 secretion is primarily driven by glucose-dependent Na^+^ uptake via SGLT-1 and intracellular glucose metabolism, which leads to closure of K_ATP_ channels and further depolarisation and exocytosis (Reimann *et al*. 2008, Gorboulev* et al*. 2012, Kuhre* et al*. 2014*a*, Sun* et al*. 2017). Additional mechanisms independent of glucose metabolism may also play a role, however, as consumption of the non-metabolised SGLT-1 substrate, 3-*O-*methylglucose, also increases plasma GLP-1 in humans (Wu* et al*. 2012). What these mechanisms are, precisely, remains unknown. Other EE cells, such as GIP-secreting K cells may also sense glucose via similar metabolism-dependent mechanisms (Parker* et al*. 2009). On the other hand, the underlying mechanisms driving glucose-induced 5-HT release remains to be determined, although 5-HT secreting enterochromaffin (EC) cells are clearly glucose-sensitive *in vitro* (Martin* et al*. 2017*b*, Lumsden* et al*. 2019) and *in vivo* (Young* et al*. 2018), and express a myriad of glucose sensors, such as SGLT1, GLUT2 and the sweet taste receptor T1R2/T1R3 (Martin *et al*. 2017*a*). Although ghrelin-secreting X/A cells demonstrate sensitivity to glucose *in vitro* (Sakata* et al*. 2012), they do not appear to directly sense luminal or vascular glucose *in vivo* (Schaller* et al*. 2003, Williams* et al*. 2003).

Fructose occurs naturally in fruit but the primary source of fructose in the modern diet is in the form of sucrose, a disaccharide, which is degraded by sucrase into glucose and fructose. Fructose transport and absorption is mediated by GLUT5, which is expressed in EC cells (Martin *et al*. 2017*a*), L cells (Reimann *et al*. 2008) and K cells (Parker *et al*. 2009). Fructose triggers the release of 5-HT (Martin *et al*. 2017*b*), GLP-1, CCK and PYY, but despite K cells expressing GLUT5, luminal fructose does not elicit *in vivo* GIP release in mice, rats or humans (Kuhre* et al*. 2014*b*). Further interrogation using a GLP-1-secreting cell line suggests that fructose-induced GLP-1 secretion is mediated by intracellular metabolism and subsequent closure of K_ATP_ channels (Kuhre *et al*. 2014*b*), although this remains to be confirmed in primary L cells. EECs also express sweet taste receptors (STRs), which comprise a heterodimer of the GPCRs T1R2 and T1R3 (Steinert* et al*. 2011, Kreuch* et al*. 2018). Genetic ablation of *T1r3* or the STR subunit, α-gustducin, significantly attenuated GLP-1 secretion upon an OGTT in mice (Jang* et al*. 2007, Kokrashvili* et al*. 2009). Although sugars are agonists for STRs, whether or not they are directly implicated in glucose-induced incretin secretion is still a subject of controversy (Gerspach* et al*. 2011, Steinert *et al*. 2011, Saltiel* et al*. 2017).

#### Proteins

Protein ingestion is a potent stimulant for the secretion of a range of gut hormones. Ingested proteins are broken down into oligopeptides and individual amino acids by proteases in the stomach. Peptides are translocated across the gut wall by the proton-coupled oligopeptide transporter PEPT1 (also known as SLC15A1), which is expressed by EECs secreting CCK (Liou* et al*. 2011*a*) and GLP-1 (Diakogiannaki* et al*. 2013). PEPT1 activity is electrogenically coupled to protons and its activation contributes to the lowering of membrane potential (Fei* et al*. 1994). PEPT1 activity is required for protein hydrolysate-induced GLP-1 secretion from murine small intestinal enteroids (Zietek* et al*. 2015) and mixed epithelial cell culture (Diakogiannaki *et al*. 2013), which underpins the reduction of endogenous glucose production by casein hydrolysates in rats (Dranse* et al*. 2018). However, the mechanisms underlying protein hydrolysate-induced I cell secretion remains to be elucidated as PEPT1 is not necessary for triggering CCK release *in vitro* (Liou *et al*. 2011*a*). Individual amino acids can also trigger the release of a range of gut hormones by activating different GPCRs. CaSR was first described as a calcium sensor and was later shown to be an amino acid sensor, preferentially activated by aromatic amino acids such as tryptophan and phenylalanine, but not branched chain amino acids (Conigrave* et al*. 2000). CCK (Liou* et al*. 2011*c*), GIP (Mace* et al*. 2012) and GLP-1 cells (Diakogiannaki *et al*. 2013) all express *CaSR* and tryptophan-induced gut hormone secretion is reliant on CaSR activity (Mace *et al*. 2012). GPR142 is implicated in tryptophan, but not protein-induced GIP and GLP-1 release (Rudenko* et al*. 2019). GIP-secreting K cells express high levels of the basic amino acid sensor *GPRC6a* (Sommer & Mostoslavsky 2014), which is activated by arginine and lysine but is insensitive to aromatic acids (Wellendorph* et al*. 2005). Intraduodenal infusion of the branched chain amino acid (BCAA) leucine stimulates the release of CCK but not other gut hormones (Steinert* et al*. 2015), while valine, another BCAA, does not stimulate CCK release (Elovaris* et al*. 2019). Emerging evidence suggests leucine-stimulated CCK secretion is mediated through the umami taste receptor T1R1/T1R3 (Tian* et al*. 2019).

Dietary protein can also regulate gut hormone biosynthesis, predominantly through the activation of mTOR, the highly conserved master regulator of cellular anabolic processes. Mice with EEC-specific deletion of TSC1, an endogenous mTOR inhibitor, had higher circulating GLP-1 levels and increased ileal proglucagon mRNA and protein levels. Elevated baseline GLP-1 is also seen with a 6-day treatment with leucine, which is associated with increased ileal proglucagon protein content. Conversely, same treatment duration with rapamycin, which potently suppresses mTOR activity, significantly decreases circulating GLP-1 levels and reversed the effects of leucine treatment (Xu* et al*. 2015). On the other hand, the biosynthesis of the orexigenic hormone ghrelin is suppressed by increased amino acid availability through increased mTOR activity in X/A cells, while rapamycin significantly increases circulating ghrelin levels (Xu* et al*. 2009, 2012). Moreover, mTOR activation also results in the downregulation of the expression of GOAT, the rate-limiting enzyme of ghrelin acylation (Muller *et al*. 2015), thereby reducing the availability of active ghrelin (Li* et al*. 2019, Mao* et al*. 2019). As such, reduced protein availability could drive hunger by increasing ghrelin levels.

#### Lipids

Dietary lipids are typically ingested in the form of triglycerides, which are broken down by pancreatic lipase into long-chain fatty acids (LCFAs) and monoacylglycerols (MAGs). These are either passively absorbed or undergo facilitated transport into enterocytes and resynthesised into triglycerides, packaged into chylomicrons and released into the lymphatic system (Iqbal & Hussain 2009). Free fatty acids signal via G-protein-coupled free fatty acid receptors (FFAR), several of which have been identified in EE cell populations and convey the ability to sense luminal short-, medium- and long-chain fatty acids. Ingestion of lipids stimulates the *in vivo* release of many gut hormones, including CCK, GLP-1 and GIP (Isaacs* et al*. 1987, Ekberg* et al*. 2016, Mandoe* et al*. 2018). Expression of the lipid amide receptor, GPR119, which is activated by monoacylglycerols, has been demonstrated in EC cells (Martin *et al*. 2017*a*), L cells (Chu* et al*. 2008), I cells (Sykaras* et al*. 2012) and K cells (Parker* et al*. 2012) and enhances the secretion of GLP-1 and GIP, but not CCK or PYY, in humans *in vivo* (Hansen* et al*. 2011). Moreover, agonists for FFAR1 and GRP119 stimulate secretion of GLP-1 (Lan* et al*. 2012, Moss* et al*. 2016) (Christensen* et al*. 2015) in various *in vitro* models, while lipid-induced GIP, CCK and GLP-1 release is substantially compromised in *Ffar1* and *Ffar4*-deficient mice (Liou* et al*. 2011*b*, Iwasaki* et al*. 2015, Sankoda* et al*. 2017). Intraduodenal lipid infusion increases the expression of FFAR1 and is positively correlated with GIP secretion, suggesting long-chain fatty acids may drive an increase in GIP secretion (Cvijanovic* et al*. 2017).

It was once thought that localisation of receptors for lipid sensing were exclusive to the apical membrane of EECs, which would allow them to directly sense luminal lipids. However, emerging evidence appears to refute this model. Vascular, but not luminal administration of the long-chain fatty acid linoleic acid and FFAR1 agonist-stimulated GLP-1 secretion in an *ex vivo* rat small intestine perfusion model (Christensen *et al*. 2015), thereby suggesting that FFAR1 resides in the basolateral membrane of GLP-1-secreting L cells. Moreover, the formation of chylomicrons appears pivotal in lipid-induced CCK (Glatzle* et al*. 2003), GLP-1 and GIP secretion (Lu* et al*. 2012, Psichas* et al*. 2017). While the immunoglobulin-like domain-containing receptor 1 has been identified as a CCK-specific chylomicron sensor (Chandra* et al*. 2013), the mechanisms controlling chylomicron-induced GLP-1 and GIP secretion remain to be elucidated, as it is not affected by the blockade of lipoprotein lipase or the inhibition of GPR119 or FFAR1 signalling (Psichas *et al*. 2017).

#### Bile acid sensing

Bile acids are amphiphilic molecules synthesized by hepatocytes from cholesterol and stored in the gallbladder. Upon exposure to lipids in the GI lumen, I cells release CCK that then triggers gallbladder contraction to release bile into the duodenum to aid the solubilization of lipids, facilitating their absorption (Lefebvre* et al*. 2009). The majority of bile acids are actively reabsorbed in the terminal ileum by apical sodium bile acid transporter and approximately 5% enters the colon, where their hydrophobicity is enhanced by microbial metabolism, thus enabling some of the bile acids to be passively absorbed while the remainder are excreted (Lefebvre *et al*. 2009). In addition to their role as GI detergents, bile acids are signalling molecules that have important implications in peripheral metabolism. The acute stimulatory effect of bile acids on EECs is mediated by the Takeda G-protein-coupled receptor 5 (TGR5), which results in elevated intracellular cAMP levels and PKA activation (Goldspink* et al*. 2018). *TGR5* expression has been confirmed in various enteroendocrine cell types, including L cells (Kuhre* et al*. 2018) and colonic EC cells (Alemi* et al*. 2013). Acute bile acid exposure stimulates the release of GLP-1 and PYY from L-cells in the small intestine and colon (Adrian* et al*. 2012, Wu* et al*. 2013*a*,*b*, Brighton* et al*. 2015, Hansen* et al*. 2016, Kuhre *et al*. 2018, Christiansen* et al*. 2019) and chronic TGR5 activation increases proglucagon biosynthesis (Harach* et al*. 2012), which may underpin the increased basal GLP-1 levels observed in TGR5 agonist-treated mice (Thomas* et al*. 2009). Bile acids have an inhibitory effect on CCK release (Koop* et al*. 1988, Koide* et al*. 1993, Marina* et al*. 2012), but it remains to be determined if this is TGR5 mediated. Bile acid sequestrants, which are anionic exchange resins that inhibits bile acid reabsorption in the terminal ileum, markedly increases GLP-1 levels in rodents in a TGR5-dependent manner (Harach *et al*. 2012). However, these effects have not been reliably translated to humans (Beysen* et al*. 2012, Smushkin* et al*. 2013, Hansen *et al*. 2016, Bronden* et al*. 2018).

Bile acid signal transduction in EECs is also carried out via the nuclear Farnesoid X receptor (FXR) (Lefebvre *et al*. 2009), which influences gene transcription pathways and the biosynthesis of gut hormones, rather than activating hormone release (Kuhre *et al*. 2018). FXR activation inhibits proglucagon biosynthesis (Jiang* et al*. 2015), thereby reducing the fasting plasma GLP-1 levels (Albaugh* et al*. 2019, Pierre* et al*. 2019). Conversely, intestinal-specific FXR inhibition results in increased intestinal proglucagon mRNA and circulating GLP-1 levels in mice (Trabelsi* et al*. 2015) but does not change circulating GIP and ghrelin levels (Pierre *et al*. 2019). The effects of FXR activation on other gut hormones remain unclear, as is the mechanisms by which intestinal FXR regulate peripheral metabolism.

### Microbial sensing

The GI tract is host to an abundance of gut microbes, or microbiota, and together with the genetic traits (collectively referred to as the gut microbiome), contribute profoundly to host metabolic processes. The importance of the gut microbiome in host metabolism has been elegantly demonstrated through the use of faecal microbiota transfer from lean and obese humans and obese mice into germ-free (GF) mice lacking a native gut microbiome, which conveys the metabolic phenotype from the donor to the host (Turnbaugh* et al*. 2006, 2009, Backhed* et al*. 2007). In addition, a core obese microbiome has been identified in humans, which contributes to obesity progression and the dysregulation of metabolism through an increase in energy harvest (Turnbaugh *et al*. 2006, 2009). It has now been established that there is bidirectional signalling between EE cells and the resident gut microbiota, which is unsurprising, considering they are constitutively in direct contact with the other (Martin* et al*. 2019).

The importance of the signalling from the microbiota to EECs is evident in reports that GF mice or broad-spectrum antibiotic-treated mice have markedly elevated circulating GLP-1 (Grasset *et al*. 2017) but reduced 5-HT (Yano* et al*. 2015). In addition to acute hormone release, the microbiota also has profound impact on gut hormone biosynthesis at the cellular level and the composition of the EE cell population. Reduced CCK and proglucagon protein expression is observed in dissociated cells from the proximal small intestine of GF mice, which was not due to reduced numbers of EE cells (Duca* et al*. 2012). Conversely, the presence of a gut microbiome acts in a chronic manner to increase the biosynthesis of 5-HT (Reigstad* et al*. 2015), contributed to by an increase in the density of 5-HT-containing cells compared to GF mice (Yano *et al*. 2015). Gut microbiota signal to EE cells through several mechanisms, including the release of microbial structural components such as lipopolysaccharides and metabolites such as short-chain fatty acids (SCFAs) and secondary bile acids (Martin *et al*. 2019).

Most mammals do not possess the enzymes to metabolise indigestible carbohydrates such as cellulose. The ability for the gut microbiota to utilize these insoluble fibre and harvest energy forms the foundation of the symbiotic relationship between host and its resident gut microbiota. Resulting metabolites can then serve as signalling molecules that regulate host metabolism or as energy substrates. Short-chain fatty acids (SCFAs) are the primary breakdown products of this process and they signal via the G-protein-coupled free fatty acid receptors (FFAR) 2 and 3 (Offermanns 2014) or by modulating nuclear histone deacetylase (HDAC) activity (Waldecker* et al*. 2008, Fellows* et al*. 2018, Larraufie* et al*. 2018). SCFA signalling through FFAR2 and FFAR3 in EE cells occurs via different secondary pathways, with each receptor having differing affinities for the dominant SCFAs: acetate, propionate and butyrate (Offermanns 2014). The expression of *FFAR2* and *FFAR3* has been identified in 5-HT-producing EC cells (Martin *et al*. 2017*a*) and GLP-1/PYY-producing L cells (Nohr* et al*. 2013). SCFAs are potent GLP-1 secretagogues (Tolhurst* et al*. 2012), stimulating L cells secretion in a region-specific manner. SCFA signalling in the small intestine occurs primarily through FFAR3, whilst FFAR2 mediates GLP-1 release from colonic L cells (Greiner & Backhed 2011). Chronic SCFA treatment increases the density of PYY-containing cells (Brooks* et al*. 2017), as well as PYY biosynthesis in a dose- and time-dependent manner in both EE cell lines and primary human colonic cells (Larraufie *et al*. 2018). On the other hand, acute exposure of primary EC cells from mice to SCFA in culture does not increase 5-HT release (Martin *et al*. 2017*b*).

In addition to indigestible carbohydrates, the gut microbiota also metabolise a range of substrates present in the intestinal lumen. Aforementioned, the intestinal flora drives the deconjugation and dihydroxylation of bile acids that escapes active reabsorption in the terminal ileum, forming secondary bile acids, which are more hydrophobic and can thus enter the enterohepatic circulation via passive diffusion (Lefebvre *et al*. 2009). As individual bile acids having different signalling profiles at TGR5 and FXR (Kuhre *et al*. 2018) and as such, the microbiota can influence gut hormone secretion by altering the composition of the bile acid pool. Microbial metabolites derived from amino acid metabolism have also been shown to modulate gut hormone secretion. While the majority of dietary proteins and amino acids are absorbed in the small intestine, small amounts of unabsorbed amino acids could go through the ileocaecal valve to enter the colon (Chung* et al*. 1979), and thus, serve as a nitrogen source for the colonic microbiota. Indole and hydrogen sulphide are metabolites produced by microbial metabolism of tryptophan and cysteine, respectively, and have both been shown to acutely stimulate GLP-1 secretion in GLUTag cells, a L cell-like GLP-1-secreting cell line (Chimerel* et al*. 2014, Pichette* et al*. 2017). Isovalerate, a volatile fatty acid derived from valine fermentation (Zarling & Ruchim 1987), has been identified as a potent EC cell secretagogue (Bellono* et al*. 2017).

The presence of the microbiota in the gastrointestinal lumen represents a rich source of microbial-associated molecular patterns (MAMPs), which are evolutionarily conserved microbial structures that are recognized by host immune cells through a range of pattern recognition receptors (PRRs). It has been speculated that EECs possess the same machinery (Bogunovic* et al*. 2007). GLP-1 secretion is markedly elevated upon LPS exposure* in vivo* in both mice (Kahles *et al*. 2014) and humans. However, emerging evidence suggests the stimulatory effect of LPS on L cells may be indirect. LPS only increased GLP-1 levels when administered systemically or in animal models of impaired intestinal barrier function (Kahles *et al*. 2014, Lebrun *et al*. 2017), implying any potential sensing machinery resides basolaterally. Importantly, LPS-induced GLP-1 increase is abolished in mice deficient in receptor for the proinflammatory cytokine IL-6 (Kahles *et al*. 2014), a known L cell secretagogue (Ellingsgaard* et al*. 2011). Together, these observations suggest that MAMP-induced gut hormone release is likely to be a reaction associated with systemic exposure, rather than to microbial structural components derived from the gut microbiota locally.

## Summary

There is a growing appreciation for the role of the gastrointestinal tract in maintaining energy and glucose homeostasis and gut-derived hormones contribute significantly to metabolic control. Enteroendocrine cells are key players in driving the metabolic functions of the gut as they release a host of different hormones in response to various luminal and vascular stimuli to elicit adequate metabolic responses, either in an endocrine manner or via the gut-brain axis through activation of extrinsic nerves. As such, enteroendocrine cells and gut hormones are in themselves, important drug targets for anti-obesity and anti-diabetes therapies and a better understanding of gut hormone physiology will greatly facilitate this process.

## Declaration of interest

The authors declare that there is no conflict of interest that could be perceived as prejudicing the impartiality of this review.

## Funding

This work did not receive any specific grant from any funding agency in the public, commercial or not-for-profit sector.
